# Variation at the DRD4 locus is associated with wariness and local site selection in urban black swans

**DOI:** 10.1186/s12862-015-0533-8

**Published:** 2015-12-11

**Authors:** Wouter F.D. van Dongen, Randall W. Robinson, Michael A. Weston, Raoul A. Mulder, Patrick-Jean Guay

**Affiliations:** Applied Ecology Research Group and Institute for Sustainability and Innovation, College of Engineering and Science, Victoria University-Footscray Park Campus, PO Box 14428, Melbourne MC, VIC 8001 Australia; Centre for Integrative Ecology, School of Life and Environmental Sciences, Faculty of Science, Engineering and the Built Environment, Deakin University, 221 Burwood Highway, Burwood, VIC 3125 Australia; Department of Zoology, University of Melbourne, Melbourne, VIC 3010 Australia

**Keywords:** Black swan, DRD4, Flight initiation distance, Habitat selection, SERT, Urbanisation

## Abstract

**Background:**

Interactions between wildlife and humans are increasing. Urban animals are often less wary of humans than their non-urban counterparts, which could be explained by habituation, adaptation or local site selection. Under local site selection, individuals that are less tolerant of humans are less likely to settle in urban areas. However, there is little evidence for such temperament-based site selection, and even less is known about its underlying genetic basis. We tested whether site selection in urban and non-urban habitats by black swans (*Cygnus atratus*) was associated with polymorphisms in two genes linked to fear in animals, the dopamine receptor D_4_ (DRD4) and serotonin transporter (SERT) genes.

**Results:**

Wariness in swans was highly repeatable between disturbance events (repeatability = 0.61) and non-urban swans initiated escape from humans earlier than urban swans. We found no inter-individual variation in the SERT gene, but identified five DRD4 genotypes and an association between DRD4 genotype and wariness. Individuals possessing the most common DRD4 genotype were less wary than individuals possessing rarer genotypes. As predicted by the local site selection hypothesis, genotypes associated with wary behaviour were over three times more frequent at the non-urban site. This resulted in moderate population differentiation at DRD4 (F_ST_ = 0.080), despite the sites being separated by only 30 km, a short distance for this highly-mobile species. Low population differentiation at neutrally-selected microsatellite loci and the likely occasional migration of swans between the populations reduces the likelihood of local site adaptations.

**Conclusion:**

Our results suggest that wariness in swans is partly genetically-determined and that wary swans settle in less-disturbed areas. More generally, our findings suggest that site-specific management strategies may be necessary that consider the temperament of local animals.

## Background

Habitat selection has important implications for the fitness of animals [1–3]. As habitats are typically heterogeneous in terms of quality, individuals should settle in areas which maximise their survival and reproductive output. Poor habitat selection decisions can lead to insidious evolutionary or ecological traps [4, 5]. The global proliferation of urban centres has modified the availability of many habitats [6]. One important consequence is that wildlife typically associated with natural environments may increasingly settle in urban areas near humans. Animals in such urban habitats may experience both advantages (e.g. less predation and increased resources [7, 8]) and disadvantages (e.g. greater human disturbance [9]) compared to those in rural areas. Generally, animals in urban areas are typically less wary of humans than their non-urban counterparts (e.g. [10–12]). The decreased wariness of urban wildlife is often attributed to learning and habituation to non-dangerous stimuli (e.g. [11, 13]). However, population differences in wariness could also be genetically determined, for instance via local selection on behaviours that are under genetic control (e.g. [12, 14]). In addition, if tolerance to humans is genetically-determined, genotype-based site selection may occur, with individuals with bold temperaments being more likely to settle in urban environments that experience higher levels of human disturbance [15–17].

The existence of consistent, individual differences in the temperament of animals (i.e. animal personalities) is well-established [18–20]. However, animal personalities may result in reduced behavioural flexibility, limiting the capacity of individuals to adapt to diverse environmental conditions. Individuals prospecting for potential breeding or feeding sites may therefore settle in habitats that best match their temperament. For example, more wary individuals may settle in areas of less human disturbance [21]. However, scant information exists on the genetic basis of temperament-based habitat selection. This is important, because temperaments may be shaped by both environmental and genetic factors [22–24]. In addition, a species’ ability to adapt to changing habitats, such as the urbanisation of natural environments, may be more constrained when temperaments are at least partly under genetic control.

In recent years, growing evidence has suggested that animal temperaments are partly genetically-determined. For example, polymorphisms at the dopamine receptor D_4_ gene (DRD4) are often associated with variation in diverse traits such as fear, novelty seeking and body mass (e.g. [25–27], but see: [28, 29]). DRD4 is an important component of the dopaminergic system, coding for a subtype of dopamine receptor in vertebrates [30]. Dopamine is a major neurotransmitter, and its modulation of the central nervous system accordingly affects diverse functions and behaviours [30]. Similarly, the serotonin transporter gene (SERT) is responsible for the transport of the neurotransmitter serotonin to neurons and has been linked to variation in diverse behaviours such as harm avoidance, anxiety, aggression and novelty seeking (e.g. [31–33], but see: [34]). Traditionally, these genes have been investigated in humans and laboratory animals. However, a growing number of studies has highlighted their importance in shaping the behaviour of wild populations of animals (e.g. [35–37]), including within-species colonisation of urban habitats. For example, differences in the frequencies of SERT alleles between urban and rural populations of common blackbirds (*Turdus melura*) suggest that genes associated with harm avoidance are under selection during urban colonisation events [29]. However, no studies have simultaneously quantified genetic and behavioural differentiation between urban and non-urban populations of animals. This information is crucial to determine whether genetic differentiation is associated with phenotypic differentiation between urban and non-urban habitats.

Here we tested for genotype- and habitat-associated differentiation in wariness towards humans of black swans (*Cygnus atratus*). We first quantified wariness in populations of swans at one urban and one non-urban wetland by estimating flight initiation distance [FID: the distance at which an escape response is initiated from an approaching pedestrian, a potentially-threatening stimulus, 17]. FIDs are known to have a substantial heritable component in other bird species (e.g. [38]). By collecting multiple FIDs for a large number of individual swans at the urban wetland, we first tested whether FID is a repeatable behaviour, a prerequisite for a heritable trait under genetic control [39, 40]. Second, we genotyped 80 individuals at the DRD4 and SERT genes to test whether wary individuals were more likely to possess certain genotypes. We then collected FID and genotypic data at the non-urban population and predicted that the frequencies of genotypes associated with increased wariness was greater in the non-urban site experiencing less human disturbance.

## Results

### Repeatability of FID

As FID is positively associated with starting distance (SD: the distance from the focal individual at which the approach commenced), we regressed FID with SD across all individuals for each trial separately. We then used the residuals for each individual (ResFID) to calculate repeatability. We detected high intra-individual repeatability in ResFID (repeatability = 0.61, F_1,131_ = 4.132, *P* = 0.044). The difference in ResFID between the two trials for each individual was not significantly related to both the days lapsed between trials (mean absolute difference in ResFID between trials - 10 or fewer days lapsed = 5.6 ± 0.8 m, more than 10 days lapsed = 6.8 ± 0.9 m; *U* = 1610.5, N_<10days_ = 54, N_>10days_ = 53, *P* = 0.263) and whether the same individual researcher approached the swan (mean difference in ResFID between trials - same researcher = 5.9 ± 0.6 m, different researcher = 7.0 ± 0.9 m; U = 1194.5, N_same_ = 94, N_different_ = 43, *P* = 0.310).

### Characteristics of DRD4 and SERT

**Fig. 1:**
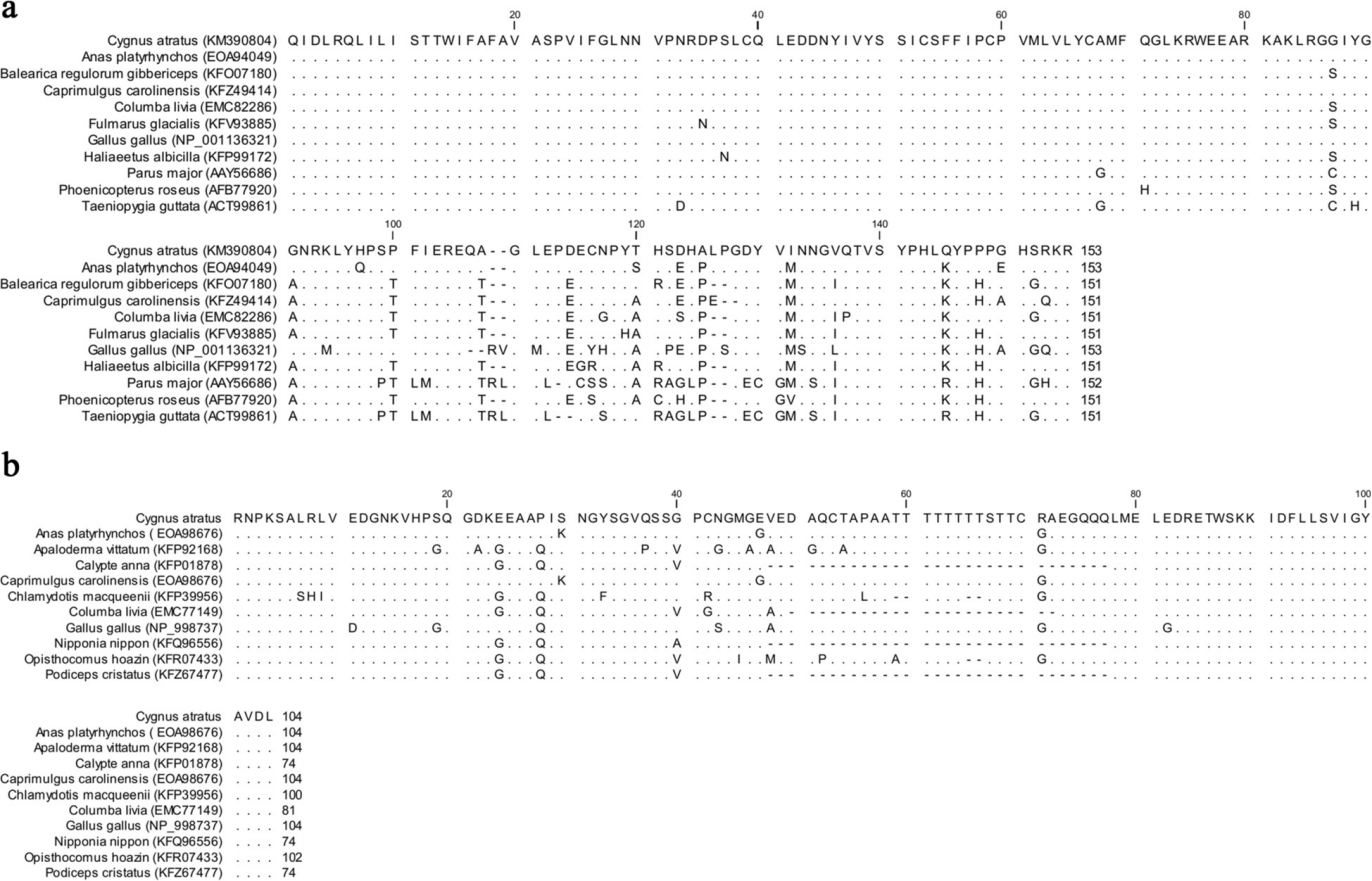
Black swan DRD4 and SERT protein alignments. Protein alignment are for (**a**) exon three of the dopamine receptor D_4_ and (**b**) the serotonin transporter, and are aligned with orthologous sequences from various bird species. Dots indicate matching amino acids relative to the black swan and dashes represent gaps. Protein sequence identifiers include the species name and GenBank accession number

The closest alignment of the swan DRD4 protein was with *Anas platyrhynchos* (E-value: 2 × 10^−104^), *Fulmarus glacialis* (E-value: 3 × 10^−98^) and *Caprimulgus carolinensis* (E-value: 3 × 10^−98^; Fig. 1a). The swan SERT gene aligned with *Anas platyrhynchos* (E-value: 2 × 10^−64^), *Gallus gallus* (E-value: 2 × 10^−48^) and *Apaloderma vittatum* (E-value: 1 × 10^−47^; Fig. 1b).

**Fig. 2:**
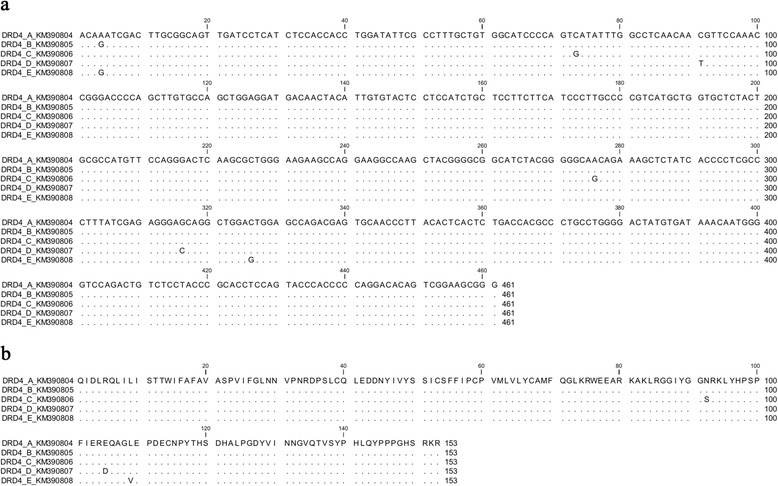
DRD4 alignment for black swans. Alignments are for (**a**) nucleotides and (**b**) amino acids of exon three of the dopamine receptor D_4_. Dots indicate matching nucleotides or amino acids relative to allele A. Allele identifiers include the allele name and GenBank accession number

All individuals were monomorphic at the SERT locus for a 335 bp allele and we identified no single nucleotide polymorphisms (SNPs) in this fragment for 24 individuals. The 461 bp fragment of DRD4 contained six variable sites, resulting in five alleles (A–E; Fig. 2a). Alleles A and B differed by a single synonymous SNP, while the remainder of alleles differed by non-synonymous SNPs (Fig. 2b). The distribution of DRD4 genotypes was highly skewed, with 83 % of individuals being homozygous at a single allele (i.e. genotype AA). The remaining individuals were either homozygous at a second allele (2 %, genotype BB) or heterozygous (15 %, genotypes AB, AC, AD, AE). Allele A was present in 98 % of individuals, while the next most common allele (B) occurred in only 8 % of individuals.

### Association between FID and genotype

**Fig. 3:**
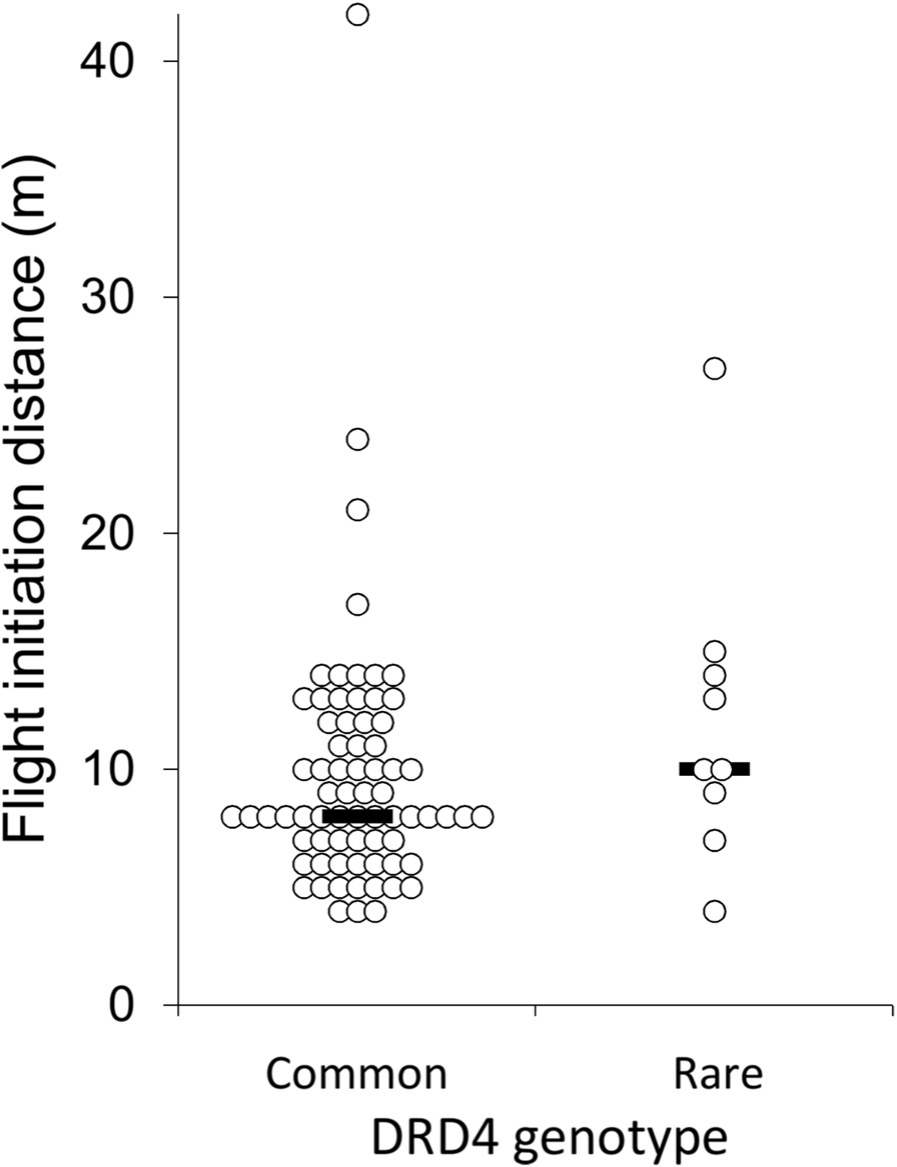
Flight initiation distances of black swans from the urban site according to DRD4 genotype. Dots represent flight initiation distances for individual swans and bars represent median values

We lacked the statistical power to test for differences in mean FID between individuals with the five genotypes that were present at the urban site. To increase the power of our analyses, we therefore categorised individuals as either having the most common genotype (i.e. AA, *n* = 71) or a rare genotype (*n* = 9). Individuals with rare genotypes had longer FIDs than individuals possessing the common AA genotype (Fig. 3; Table 1). In addition, flight initiation distances were strongly positively related to starting distance, but not the focal individual’s distance from water or microsatellite heterozygosity (percentage change in deviance = 14.5 %; Table 1).

**Table 1:** Factors associated with flight initiation distances in black swans. DRD4 genotype (i.e. common or rare genotype), heterozygosity at eight microsatellite loci, starting distance and distance from water were included as independent variables in generalised linear mixed models, incorporating swan identity as a random factor (random effect variance = 0.092 ± 0.033, Z = 2.757, *P* = 0.006)

Predictor variable	F_1,464_	*P*
DRD4 genotype	5.192	**0.023**
Microsatellite heterozygosity	1.296	0.255
Starting distance	119.022	**<0.001**
Distance from water	1.041	0.308

Significant effects are highlighted in bold

### Population differences in FID and DRD4 genotypes

**Fig. 4:**
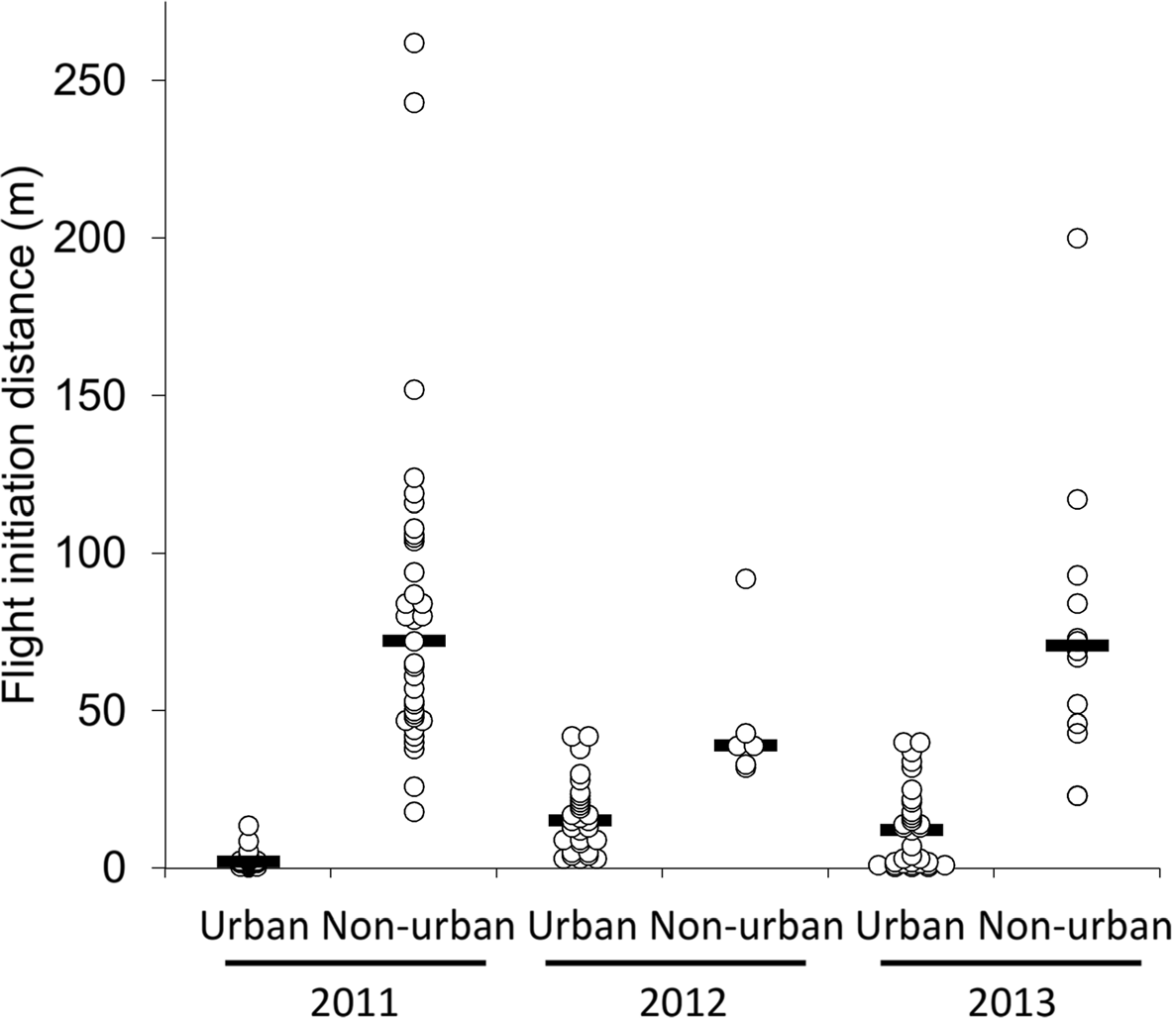
Flight initiation distances of black swans between urban and non-urban swans, separated by year. Dots represent flight initiation distances for individual swans and bars represent median values

Black swans were more wary at the non-urban site than at the urban site (mean starting distance - urban site = 39 ± 2.5 m, non-urban site = 121 ± 12.0 m; generalised linear model: population - Wald χ^2^ = 64.477, d.f. = 1, *P* < 0.001; year - Wald χ^2^ = 18.269, d.f. = 2, *P* < 0.001; location*year - Wald χ^2^ = 35.579, d.f. = 2, *P* < 0.001; starting distance - Wald χ^2^ = 12.999, d.f. = 1, *P* < 0.001: Fig. 4). Overall, the mean flight initiation distance was 13 ± 0.4 m at the urban site and 96 ± 15.9 m at the non-urban site.

Forty percent of swans from the non-urban site possessed a rare genotype, compared with only 11.2 % from the urban site (Fisher’s exact: *p* = 0.005: Table 2). Accordingly, we detected moderate genetic population differentiation at the DRD4 locus (F_ST_ = 0.080, *P* <0.001). The F_ST_ based on microsatellite loci indicated low neutral genetic differentiation between the urban and non-urban sites (F_ST_ = 0.015, *P* = 0.002).

**Table 2:** Abundance (%) of the five DRD4 genotypes found in the urban and non-urban populations

	Population	
Genotype	Urban	Non-urban
	(*n* = 80)	(*n* = 20)
AA	88.8	60.0
AB	3.8	15.0
AC	0.0	10.0
AD	5.0	0.0
AE	1.3	10.0
BB	1.3	5.0

## Discussion

The repeatability estimate of flight initiation distances in black swans was high and very similar to previous estimates in other species (e.g. [35, 38]). As repeatability in a trait sets the upper limit for its heritability [39, 40], this suggests that FIDs may have a substantial heritable component. In support of this, we have shown here that wariness in black swans is associated with polymorphisms at the DRD4 gene. The 83 % of individuals that possessed the most abundant AA genotype displayed shorter flight initiation distances than swans possessing rarer genotypes. In contrast, we found no variation in the length of exonic codon repeats located within the SERT gene. This contrasts with a previous study on common blackbirds, which detected low levels of interpopulational variation at the SERT gene but at a much larger geographic scale than in the present study [29]. We found clear population differences in FIDs, with swans from the urban wetland initiating flight 83 m closer than those sampled at the non-urban wetland, where less human disturbance occurs. Finally, we detected moderate differentiation at the DRD4 gene despite the sites being separated by only 30 km, a relatively short distance for this highly-mobile species [41]. This pattern arose due to the greater proportion of swans at the non-urban site possessing rare DRD4 genotypes, which were associated with greater wariness. Although population differences in wariness to humans may arise due to multiple factors, our data suggest that they may also, at least in part, be explained by genotype-based site selection.

As the data for this study were collected from the urban and non-urban sites in different years, an effect of year on site differences in FID could not be excluded. However, it is unlikely that year differences could have driven the results reported here. First, the greater wariness of non-urban birds is well-documented [17] and the differences in wariness of the two swan populations appears to be temporally stable. In addition, a long-term capture study at the urban site [42, 43] suggests that at least this population is largely resident and, as such, significant reversals of DRD4 allelic frequencies are unlikely.

We do not know how variation at the DRD4 gene translates into differences in wariness in swans. SNPs detected in this study presumably alter the functionality of the dopamine receptor D4 and its affinity to dopamine. In turn, the modulation of dopamine signalling in the brain regulates multiple traits, including fear (e.g. [26]). The pleiotropic effects of DRD4 suggest that swans with different genotypes may also vary in other traits, such as exploratory behaviour (e.g. [37, 44]), novelty seeking (e.g. [35]) or body condition (e.g. [36]). Alternatively, linkage disequilibrium may result in the SNPs detected in this study being linked to SNPs at other genes which also regulate fear, such as the SERT gene.

The high frequency of the AA genotype, which was associated with the shortest FIDs, suggests that selection may favour the AA genotype in the study populations. This is despite the advantages of displaying high levels of wariness towards potential predators [45]. Selection may favour shorter FIDs when increased vigilance and wariness is associated with increased energetic costs, such as the reduction of foraging time or increase in energetic expenditure during flight responses. For example, individual Iberian wall lizards (*Podarcis hispanica*) which habituate more rapidly to a non-threatening stimulus, increase their body condition (a correlate of fitness in the species) more rapidly than individuals that habituate less [46].

The tendency of animals to be less wary around humans in areas experiencing high human traffic is a common pattern and may be related to various non-mutually exclusive factors. For example, habituation may occur when animals experience a repeated benign stimulation, resulting in a decrement in a certain behaviour, unrelated to sensory or motor fatigue [47]. An untested possibility is that swans at the urban site may have short flight initiation distances because humans are a very common and non-threatening stimulus at the site. Indeed, the difference in FIDs between swans with common and rare genotypes (8 vs 14 m, respectively) was relatively small compared to the difference in FIDs between the urban and non-urban populations (13 vs 96 m, respectively). This suggests that in addition to a possible effect of DRD4 variation on wariness, other factors, such as habituation to humans, contributed to site differences in wariness.

Second, these patterns may also arise due to local adaptations, with selection favouring certain genotypes at each site and little population mixing. However, the close proximity of the sites suggest that at least some migration between the sites occurs. This is further supported by the low, but possibly biologically-meaningful, genetic differentiation at microsatellite loci. This reduces the likelihood of site-specific adaptations evolving. For example, between 2010 and 2012, eleven neck-collared swans captured at the urban site were observed at the non-urban site, at a distance of between 700 m and 84 km from the site of their previous sighting (mean = 27.7 ± 24.6 SD km; Mulder, unpublished data). Generally, one migrant per generation is sufficient to eliminate any population genetic differentiation [48], suggesting that local adaptation is not operating in these populations to a substantial degree. However, population differentiation at the neutral loci was significant, despite likely migration between the populations. Lastly, local site selection may be occurring. Black swans display strong inter-populational seasonal movements, which are related to various factors such as rainfall and stream flow [49]. The increased wariness of swans at the non-urban site, coupled with the higher frequency of DRD4 genotypes associated with wary behaviour at this site, suggest that more wary individuals settle in habitat with lower human usage. In contrast, bolder individuals may be more likely to settle at the urban site due to the greater resource abundance, including frequent anthropogenic feeding (van Dongen, personal observation), or a potentially lower predation risk compared to rural areas [e.g. 8].

## Conclusions

We have shown here that the fear of humans by black swans may be partly genetically-determined. Our findings also suggest that, in addition to learning or habituation, the lower responsiveness of wildlife in urban areas may be related to temperament-based local site selection. Our findings have important implications for conservation biology. First, the estimation of FIDs of threatened or sensitive species is an important tool used by conservation managers interested in creating buffer zones around sensitive animal habitat [17]. However, within-species FID estimates can be highly labile and are associated with multiple factors associated with both the stimulus, focal individual and local environmental conditions [12, 35, 50, 51]. Our findings that individual FIDs may be partly genetically-determined, coupled with the high repeatability of FIDs, suggests that inter-individual variation in FID is greater than intra-individual variation. This justifies the estimation of FIDs of specific individuals based on single, or a small number of, approaches. In addition, population differences in DRD4 genotype frequencies may lead to variability between sites in wariness to humans and hence the need for site-specific buffer zone sizes. The introduction of higher levels of human presence at previously undisturbed sites, such as when urban development occurs around wetlands or improved human access is provided, is likely to displace individuals who are more responsive to human presence. This is likely to effectively introduce local selection. Finally, our findings that certain animals may be able to cope better in heavily disturbed habitats have important implications for captive breeding programs of threatened species that will eventually be released in close proximity to humans. Individuals earmarked for reintroduction may be selected for specific temperaments more conductive to survival in the wild [1].

## Methods

### Field work

#### Study sites

Field work was conducted at two wetlands in Victoria, Australia, with contrasting visitor regimes: Albert Park Lake (APL; 37°50’S, 144° 58’E), an urban recreational park, which receives around 5 million visitors per annum [43, 52], and the Western Treatment Plant, Werribee (WTP; 37°54’S, 144° 40’E), a restricted-access wastewater processing site in a non-urban environment [53]. Black swans at APL exhibit relatively low responsiveness to humans [54], while those at WTP appear to be more wary [17]. The two sites are separated by about 30 km. No hunting occurs at either site. The usual breeding season of this species extends from winter to spring [55].

Black swans were captured at APL between 2006 and 2013 and at WTP between November and December 2004. Birds were captured by hand and fitted with a metal leg band at WTP, and a metal leg band and a neck collar displaying a unique identification code at APL [42, 56]. Blood samples were taken from the tarsal vein for subsequent genetic analysis.

Flight initiation distances of swans at both sites were measured throughout the entire year between 2011 and 2013. To test whether polymorphisms at DRD4 and SERT were associated with flight initiation distances, we collated FIDs of neck-collared swans at the urban site. In addition, to test for population-level differences in FIDs between the urban and non-urban sites, we collected FIDs from any swan encountered at the non-urban site, regardless of whether we had previously captured the swan. Due to the large swan population at WTP, and our focus on comprehensive sampling at APL, we were unable to collect FID data on known swans at WTP for which we had a genetic sample.

Flight initiation distances were collected at both sites using methods outlined in detail elsewhere [51, 54, 57]. Briefly, we made standardised approaches to birds foraging on land. FIDs are positively associated with starting distance [58, 59], so we recorded SD for each approach. In addition, we recorded the distance of the focal individual from the edge of the shoreline because FIDs may be longer at greater distances from the nearest refuge [51]. An approach was made by identifying an individual that was foraging on land and walking directly towards it at a slow pace (approximately 1 ms^−1^). The distance from the researcher at which the swan initiated an escape response, either by walking, flying or swimming away, was recorded as the flight initiation distance. All distances were measured with an accuracy of one metre using a Bushnell Elite 1500 range finder. Multiple researchers collected FID data at each site, however inter-researcher differences in estimation of flight initiation distances of swans is low [54].

Up to 23 FIDs were collected per swan at the urban site (mean = 6.1 ± 0.5 SE FIDs/individual), while only one FID was likely collected per bird at the non-urban site. Only adult swans were included in this study. To ensure that we did not resample the same swan at the non-urban site, we monitored the location of individual birds that had already been sampled. In addition, the high abundance of the species at the site minimised the likelihood that the same swans were sampled on multiple days or years. When the focal individual was located within a flock of birds, we did not resample any of the other individuals within that flock. Flock size is known not to influence individual FIDs in this species [51].

Fieldwork was conducted under the following permits: Victoria University Animal Ethics Committee Permit AEETH 15/10, Deakin University Animal Ethics Committee Permits A48/2008 and B32/2012, the University of Melbourne Animal Ethics and Experimentation Committee of the Faculty of Science (register no. 0705887.4), DSE Scientific Permits, 10004585, 10004656 and 10005536 and a Western Treatment Plant Study Permit SP 08/02.

### Genetic analysis

#### DRD4 and SERT genotyping

DNA was extracted from blood samples using the salting-out procedure [60]. We amplified 461 bp of the DRD4 gene using the primers F1-E3-DR4D (5’-CCRCTSAACTACAACCGGCG-3’) and R1-E3-DR4D [5‘-YTCCCGGCCGTTGATCTTGG-3’: 36]. We additionally amplified an exonic trinucleotide codon repeat in the SERT gene using the 6-FAM-labelled Sert_Ex1m_F2 primer (5‘-ATCTCCACACATTYCCCAGA-3’) and Sert_Ex1m_R2 [5‘-AGGAACCCTAAATCTGCCCTAC-3’: 29]. Variation in the repeat number of this codon has previously been shown to correlate with individual differences in harm avoidance behaviour [29]. We therefore quantified the length of this fragment of all individuals. We additionally sequenced the SERT gene for 24 individuals to test for the presence of SNPs.

PCR was performed in 15.0 μl reaction volumes containing the forward and reverse primer (1.1 mM each), 0.05 units of GoTaq DNA polymerase (Promega), 1x Colorless GoTaq Flexi Buffer, 3.3 mM MgCl_2_ (Promega), 0.2 mM dNTPs and approximately 50 ng of genomic DNA. PCRs were run on a BioRad Mycycler Thermocycler. For DRD4, an initial denaturation step (95 °C, 3 min) was followed by 35 cycles of 45 s at 95 °C, 1 min at 60 °C, 1 min at 72 °C, and a final extension step for 5 min at 72 °C. DRD4 fragments were commercially sequenced (Australian Genome Research Facility) in both directions and viewed in CLC Main Workbench 7.0.3 (CLC Bio). Only SNPs that were present in both the forward and reverse sequence for each individual were considered genuine. All others were assumed to be sequencing artefacts. Following [36], we then identified unique alleles that differed from all other alleles by at least one SNP and assigned genotypes to each individual based on the identity of their two alleles. The unique DRD4 alleles generated for this study were then submitted to GenBank [GenBank: KM390804-KM390808].

For SERT, an initial denaturation step (94 °C, 5 min) was followed by 35 cycles of 30 s at 94 °C, 60 s at 53 °C, 60 s at 72 °C, and a final extension step for 15 min at 72 °C. Fragment analysis of the SERT codon repeats was also conducted commercially (Australian Genome Research Facility) and results were viewed in GeneMarker 2.6.3 (SoftGenetics LLC). The SERT locus of 24 individuals was also commercially sequenced in both directions (Australian Genome Research Facility).

We confirmed correct amplification of the DRD4 and SERT genes via a BLASTP search [61] of the swan protein sequences in GenBank. The strength in similarity between protein sequences was assessed via the E-values, with values closer to zero corresponding to greater sequence similarity [62].

#### Microsatellite genotyping

To control for heterozygosity-fitness correlations [63] that may result in artefactual associations between gene polymorphisms and FIDs, we also genotyped birds at eight microsatellite loci that were presumably neutrally-selected, including Cam3 and Cam9 [64], TTUCG5 [65], TSP.1.20.9 and TS.1.29.32 [66], Caudo24 [67]. We additionally amplified the Cam4 and Cam10 loci as described in [64], but with one modified primer per locus to increase the length of the amplified fragment (modified primers - Cam4L reverse primer: 5‘-CCAAACCACTTACAACCATTG-3’; Cam10L forward primer: 5‘-M13-AATGGCAGTGGAATACAAAG-3’). PCR was performed for each locus as described in the above references and in [68]. Fragments were electrophoresed on a Beckman Coulter 8000XL automated sequencer. Fragment sizes were scored using an automated binning system in the Beckman Coulter 8000XL fragment analysis software, which were also confirmed visually. This binning system is well-established for these loci and has previously been used elsewhere for this species [64, 68]. We confirmed that all loci were under Hardy-Weinberg equilibrium using Cervus 3.0 [69].

### Statistical analyses

Analyses on the repeatability of FIDs, and the association between FID and gene variation were conducted using data collected from APL alone. Analyses on population differences in FID, and in population genetic differentiation, were conducted using data from both APL and WTP.

Repeatability in flight initiation distances was estimated following [40], where repeatability is given by $r=\frac{{S^2}_A}{{S^2}_W+{S^2}_A}$ (S_A_ is the among-groups variance component and S_W_ is the within-group variance component). We selected 105 swans for this analysis, for which we had two FID estimates for each individual that were collected within a few months of each other (mean time lapsed between first and second trial = 18.2 ± 1.8 SE days, 1–93 days). As FID is positively associated with SD, we first regressed FID with SD across all individuals for each trial separately. We then used the residuals for each individual (ResFID) for the repeatability analysis. Thus, an individual with a positive residual had a longer FID than expected from its SD. We then tested whether the absolute difference in ResFID between the first and second trial correlated with time lapsed. We did not expect a linear relationship between FID difference and days lapsed, but instead expected more similar FIDs when less time had passed between FID estimates. The distribution of days lapsed was approximately bimodal, with a median of 10 days. We therefore tested whether the absolute difference in ResFIDs was greater for birds sampled more than 10 days apart. Similarly we tested whether the absolute difference in ResFID between trials varied according to whether the same researcher or two different researchers collected the two FID estimates for each individual.

As we found no interindividual variation at the SERT codon repeats, no further analyses were conducted for this gene. We translated all DRD4 alleles into protein sequences using CLC Main Workbench 7.0.3 (CLC Bio) to test whether alleles differed in synonymous or non-synonymous base substitutions. However, for downstream analyses, we characterised differences between individuals based on genotypes and not protein sequences as synonymous base-pair substitutions may also result in changes in protein functionality [70]. In addition, synonymous substitutions may be associated with non-synonymous SNPS within other regions of the DRD4 gene (e.g. [44]).

For our analysis on the association between DRD4 genotypes and FID, we often had multiple estimates of FID for each individual. As FID may vary with multiple factors (e.g. SD and distance to the nearest refuge), we did not average multiple estimates per individual. Instead we included all FID data for all individuals and conducted generalised linear mixed modelling, incorporating swan identity as a random effect and FID as the dependent variable. As FID followed a gamma distribution for both populations and allele cohorts (i.e. ‘common’ vs ‘rare’ alleles), we used a log link for our analyses. Mixed models are additionally useful as they can be used for data with heterogeneous variances between groups [71, 72]. As the uneven distribution of alleles resulted in unequal samples sizes in our analyses on the effect of DRD4 genotype on FID, the possibility remains that certain individuals with rare alleles and extreme FIDs may bias the results. To minimise these effects, we grouped all rare alleles into one category to increase the robustness of the analysis (as opposed to treating each allelic variant as a separate group). Variances between these two groups were homogenous (Levene’s test: F_1,478_ = 0.032, *P* = 0.858). Models include DRD4 genotype (i.e. ‘common’ or ‘rare’), starting distance, the distance to the nearest refuge and microsatellite heterozygosity as predictor variables. Lastly, to quantify how well the model fitted the data, we calculated the percentage change in deviance between the focal model and the model containing only the intercept [73, 74].

To test for population differences in FID, we only included one randomly-selected FID estimate per individual swan. The analysis included 53 FIDs from the non-urban site and 93 from the urban site. Finally, genetic differentiation between populations was estimated at the DRD4 locus and neutral microsatellite loci by conducting an analyses of molecular variance in ARLEQUIN 3.5.1.2 [75]. Although F_ST_ values cannot be directly compared between neutral microsatellites and other genetic loci [76], their quantification can still provide information on the degree of genetic differentiation between the two sites.

We conducted all non-genetic statistical analyses using SPSS 20.0 (SPSS, Chicago, Illinois, USA). Non-parametric tests were used when the assumption of data normality or homoscedasticity was not met. All data are presented as mean ± SE. For GLMMs, we present predicted means ± SE.

## Abbreviations

APL: Albert Park Lake
DNA: Deoxyribonucleic acid
dNTPs: Deoxynucleotide triphosphates
DRD4: Dopamine receptor D_4_
FID: Flight initiation distance
MgCl_2_: Magnesium chloride
PCR: Polymerase chain reaction
ResFID: Residual flight initiation distance
SD: Starting distance
SE: Standard error
SERT: Serotonin transporter
SNP: Single nucleotide polymorphism
WTP: Western Treatment Plant
